# Lipoprotein(a) as a risk factor for atherosclerotic cardiovascular disease in patients in non-metropolitan areas of Brandenburg, Germany

**DOI:** 10.3389/fcvm.2024.1302152

**Published:** 2024-03-07

**Authors:** Philipp Hillmeister, Kangbo Li, Mengjun Dai, Mesud Sacirovic, Nikolaos Pagonas, Oliver Ritter, Peter Bramlage, Anja Bondke Persson, Ivo Buschmann, Claudia Zemmrich

**Affiliations:** ^1^ Department for Angiology, Center for Internal Medicine I, Deutsches Angiologie Zentrum (DAZB), University Clinic Brandenburg, Brandenburg Medical School (MHB) Theodor Fontane, Brandenburg/Havel, Germany; ^2^ Faculty of Health Sciences, Joint Faculty of the Brandenburg University of Technology Cottbus—Senftenberg, The Brandenburg Medical School Theodor Fontane and the University of Potsdam, Brandenburg Medical School Theodor Fontane, Brandenburg an der Havel, Germany; ^3^ Charité—Universitätsmedizin Berlin, Berlin, Germany; ^4^ Department for Cardiology, Center for Internal Medicine A, University Clinic Ruppin-Brandenburg, Brandenburg Medical School (MHB) Theodor Fontane, Neuruppin, Germany; ^5^ Department for Cardiology, Center for Internal Medicine I, University Clinic Brandenburg, Brandenburg Medical School (MHB) Theodor Fontane, Brandenburg an der Havel, Germany; ^6^ Institute for Pharmacology and Preventive Medicine, Cloppenburg, Germany

**Keywords:** Lipoprotein (a), risk factor, atherosclerotic cardiovascular disease, non-metropolitan area, Germany

## Abstract

**Background and aims:**

In the non-metropolitan region of Brandenburg (Germany), which is characterized by high rates of cardiovascular diseases and underserved medical care, there is a lack of awareness regarding lipoprotein(a) [Lp(a)] as a risk factor. In addition, data from patients with atherosclerotic cardiovascular disease (ASCVD) in diverse regional backgrounds, including the understudied Brandenburg cohort, and various healthcare statuses remain insufficient.

**Methods:**

In this WalkByLab study, Lp(a) levels were monitored in a non-metropolitan cohort (*n* = 850) in Brandenburg, Germany, comprising 533 patients at high cardiovascular risk and 317 healthy controls. Patients underwent a comprehensive angiological screening, which included blood serum analysis, assessment of medical and family history, cardiovascular risk, and disease status, and evaluation of lifestyle and quality of life. All parameters were evaluated with regard to two groups based on Lp(a) levels: low (<50 mg/dl) and high (≥50 mg/dl).

**Results:**

Brandenburg patients with cardiovascular diseases showed higher Lp(a) levels than healthy controls (24.2% vs. 14.8%, *p* = 0.001). Logistic regression analysis with different characteristics revealed that Lp(a) was an independent risk factor significantly associated with ASCVD (OR 2.26, 95% CI 1.32–3.95, *p* = 0.003). The high-Lp(a) group showed a higher proportion of patients with coronary artery disease, peripheral artery disease, or cerebrovascular disease compared to the low-Lp(a) group (50% vs. 36.8%; 57.7% vs. 45.8%; 17.6% vs. 9.2%; *p* = 0.004); also, a higher percentage of patients in the high-Lp(a) group had heart failure (72.8% vs. 53.2%, *p* = 0.014) and myocardial infarction (24.7% vs. 13.9%, *p* = 0.001). The high-Lp(a) group exhibited higher rates of statins (63.1% vs. 50.4%, *p* = 0.003), ezetimibe (14.8% vs. 5.5.%, *p* = 0.001), and beta-blockers (55.7% vs. 40.7%, *p* = 0.001) use. Lp(a) levels were found to be independent of physical activity or smoking behavior and did not change over time (12 months).

**Conclusions:**

Our study highlights the significance of elevated Lp(a) levels in Brandenburg cardiovascular patients and identifies them as an independent risk factor for ASCVD, which has implications for addressing cardiovascular health of non-metropolitan populations.

## Introduction

Hyperlipidemia is a major risk factor for cardiovascular disease (CVD) (1); however, clinical strategies have mostly focused on lowering plasma low-density lipoprotein (LDL) cholesterol levels as a therapeutic measure (2). Currently, lipoprotein a [Lp(a)] is gaining importance in medical research and is increasingly recognized as a cardiovascular risk factor (3). Since 2019, the European Society of Cardiology (ESC) and the European Atherosclerosis Society (EAS) guidelines for the management of dyslipidemia give a class IIa recommendation in measuring Lp(a) levels at least once in every adult's lifetime to identify individuals with congenitally elevated Lp(a) levels (4). Here, the EAS statement from 2022 confirmed that an Lp(a) threshold of 50 mg/dl should be considered a “risk enhancer” to determine an individual's estimated 10-year risk score for atherosclerotic cardiovascular disease (ASCVD) (5).

However, a global perspective reveals significant variations in mean Lp(a) concentrations across different populations, with sequential increases observed in Chinese, Caucasian, South Asians, and Blacks (16, 19, 31, and 75 nmol/L, respectively) (6, 7). Given this diversity, it becomes imperative to investigate whether the established threshold of 50 mg/dl is applicable across cohorts from various regions. Therefore, the EAS statement from 2022 expressed the need for data from patients with different regional backgrounds, as the serum Lp(a) level exhibits large variations between individuals and ethnicities. Furthermore, the EAS statement emphasizes difference in healthcare service availability between urban and non-urban populations, which further supports the value of a stable and strong risk marker such as Lp(a) (8). Aligning with the 2022 EAS statement, this study is dedicated to elevate awareness of Lp(a) and its connection to morbidity characteristics specifically in the cohort in Brandenburg, Germany.

This Brandenburg cohort represents individuals in an area with one of the highest rates of cardiovascular diseases in Germany and an area that is medically underserved, particularly for the elderly (9). Here, Lp(a) will be comprehensively investigated in a specific homogeneous elderly German population residing in the non-metropolitan region (this region is primarily rural, lacks large urban centers, features small towns or villages, and has an elderly population) of Brandenburg (10). The primary focus is to unravel the intricate role of Lp(a) in the development of atherosclerosis, particularly examining its effects on the vasculature, a dimension that has not been conclusively determined in previous research (11, 12). Hence, in this study, we introduce a novel dimension by investigating the role of Lp(a) in the development of peripheral artery disease (PAD), coronary artery disease (CAD) and cerebrovascular disease (CeVD) independently of other risk factors, with a specific emphasis on its occurrence within the unique cohort of Brandenburg, Germany. Remarkably, among the variables used to assess cardiovascular risk, such as blood glucose, cholesterol, body mass index (BMI), or endothelial function, Lp(a) stands out as a relatively stable parameter resistant to significant alterations induced by changes in lifestyle, medical treatment, and diet (13). Compelling evidence suggests that Lp(a) levels may undergo changes during childhood, posing intriguing questions about the dynamic nature of this lipoprotein across different life stages (14). While adult Lp(a) levels are generally assumed to remain relatively constant, the lack of valid follow-up studies on this subject prompts a need for a more comprehensive investigation within the specific demographics of the Brandenburg cohort.

To achieve this goal, this study seeks to investigate Lp(a) within an ASCVD cohort in the federal state of Brandenburg, Germany, which has been underexplored and undertreated. Starting with the baseline, we conduct a 1-year follow-up, which includes a subsequent assessment to observe changes in Lp(a) levels over the course of the year.

## Materials and methods

### WalkByLab

The WalkByLab (www.walkbylab.com) is a clinical trial conducted at the University Clinic Brandenburg, Brandenburg Medical School, which aims to investigate patients at risk of cardiovascular diseases. A multimodal standard was applied to longitudinally assess cardiovascular function and angiological parameters in healthy individuals at risk and patients with ASCVD.

### Study objectives

The primary objective of this study was to investigate Lp(a) levels in 850 participants stratified based on their cardiovascular risk status. Participants were classified into low-Lp(a) and high-Lp(a) groups according to the 2022 EAS consensus statement (5), which set the threshold for high Lp(a) at ≥50 mg/dl (5).

### Patient population

Patients were included in the study as soon as they provided written informed consent. Exclusion criteria were a life expectancy of less than 1 year or the inability to follow the written informed consent or physician's instructions. High-risk cardiovascular patients were defined based on the following criteria: being aged ≥18 years, having pre-existing PAD or CAD, which was defined as ≥50% coronary (angiographic) or peripheral arterial (aortic, infra-aortic, or carotid) stenosis (angiographic or duplex ultrasound), or having a history of percutaneous coronary intervention. All parameters were collected at baseline and during a 12-month follow-up visit. For a full description of the study design, see also Zemmrich et al. (10).

### Data collection

The patients’ current cardiovascular status was obtained, as along with an assessment of risk factors and a history of concomitant diseases. Medication usage, correlation of risk factors, lifestyle factors, and cardiovascular events were investigated based on Lp(a) groups, with a focus on ASCVD, particularly CAD and PAD. Weekly physical activity history, smoking habits, alcohol consumption, and health-related quality of life (QoL) was recorded by means of a questionnaire using the Short Form 36 (SF-36) and the Pain Disability Index (PDI).

### Laboratory analyses

Laboratory analyses included the following parameters: complete blood count, Lp(a), total cholesterol, LDL-C, high-density lipoprotein cholesterol (HDL-C), triglycerides, blood glucose, estimated Glomerular Filtration Rate (eGFR), and hemoglobin A1C.

For the Lp(a) measurement, the Tina-quant Lipoprotein (a) Gen. 2 kit (Roche Diagnostics GmbH) was used and evaluated via the Cobas 6000 automated measuring system (unit of measurement: mg/dl).

### Statistical analysis

The primary biometric analysis of all collected data is descriptive. Patients were divided into subgroups using an Lp(a) threshold of 50 mg/dl (reference/rationale). Results are presented in tabular and graphical forms based on the predefined analysis plan. For normally distributed continuous variables, the number of patients, mean, and standard deviation (SD) were calculated, and groups were compared by *t*-tests; for non-normally distributed continuous variables, the medians and quartiles were computed with comparisons done by Wilcoxon tests; for categorical variables, frequencies and percentages were determined and compared by Fisher's exact test; standard Bland–Altman plots were used to show the Lp(a) delta difference and concordance/agreement level; additionally, a logistic regression model was used to analyze the independence of the risk factors.

The relationship between baseline characteristics and changes in Lp(a) is explored by comparing extreme groups with either an increase or a decrease of more than two standard deviations, with the excluded smaller changes treated as rather random variations.

Statistical analyses were conducted using R 4.2 [R Core Team (2023). R: A language and environment for statistical computing. R Foundation for Statistical Computing, Vienna, Austria. https://www.R-project.org/].

## Results

### Patient characteristics

Among the 850 participants, 674 showed an Lp(a) level below the 50 mg/dl threshold with a median of 4 mg/dl (25th/75th percentile; 3/38), whereas 175 patients demonstrated an Lp(a) level equal to or above the 50 mg/dl threshold with a median of 83 mg/dl (64/104) (Table 1). Sex, BMI, systolic blood pressure, diastolic pressure, as well as LDL-C, triglyceride, and eGFR, did not differ between the low-Lp(a) and high-Lp(a) groups. Furthermore, the high-Lp(a) group was significantly associated with a lower median ankle–brachial index (ABI) of 0.9 (0.8/1.0) compared to the low-Lp(a) group with an ABI of 1.0 (0.9/1.0). The high-Lp(a) group also had a lower physical functioning score of 55 (40/80) and a higher pulse wave index (PWI) of 165 (122/245) compared with the low-Lp(a) group, which had a physical function score of 65 (45/85) and a PWI of 140 (108/200).

**Table 1:** Patient demographics and characteristics in the low-Lp(a) and high-Lp(a) groups.

	Overall	Low-Lp(a) group	High-Lp(a) group	*p*
Male sex	489	393 (58.3%)	96 (54.5%)	0.392
Female sex	361	281 (41.7%)	80 (45.5%)	
Age, years	67 (61/75) [*n* = 850]	67 (60/75) [*n* = 674]	67 (61/76) [*n* = 176]	0.655
Lp(a), mg/dl	7 (3/38) [1 → 257] [*n* = 850]	4 (3/10) [1 → 50] [*n* = 674]	83 (64/104) [50 → 257] [*n* = 176]	0.001
BMI, kg/m^2^	28 ± 5 [14 → 65] [*n* = 803]	28 ± 5 [14 → 65] [*n* = 632]	28 ± 5 [18 → 45] [*n* = 171]	0.989
Systolic blood pressure, mmHg	141 ± 18 [91 → 220] [*n* = 838]	141 ± 18 [91 → 220] [*n* = 663]	139 ± 19 [97 → 204] [*n* = 175]	0.114
Diastolic blood pressure, mmHg	79 ± 11 [47 → 149] [*n* = 836]	80 ± 11 [47 → 149] [*n* = 661]	78 ± 10 [50 → 111] [*n* = 175]	0.129
LDL-C, mmol/l	85 (64/111) [22 → 347] [*n* = 480]	86 (64/111) [22 → 347] [*n* = 363]	83 (65/109) [24 → 230] [*n* = 117]	0.458
Triglyceride, mmol/l	119 (88/176) [35 → 2,328] [*n* = 480]	119 (88/179) [35 → 2,328] [*n* = 363]	110 (87/170) [42 → 398] [*n* = 117]	0.195
eGFR, mL/min/1.73 m2	76 (60/91) [10 → 134] [*n* = 480]	75 (59/91) [10 → 125] [*n* = 365]	79 (63/91) [22 → 134] [*n* = 115]	0.295
ABI	1.0 (0.9/1.0) [0.3 → 1.5] [*n* = 846]	1.0 (0.9/1.0) [0.3 → 1.5] [*n* = 671]	0.9 (0.8/1.0) [0.4 → 1.5] [*n* = 175]	0.021
PWI	144 (110/207) [1 → 1,000] [*n* = 846]	140 (108/200) [1 → 1,000] [*n* = 671]	164 (122/245) [65 → 1,000] [*n* = 175]	0.001
General health SF-36	50 (40/62) [0 → 95] [*n* = 825]	50 (40/65) [0 → 95] [*n* = 653]	50 (40/60) [10 → 95] [*n* = 172]	0.138
Physical functioning SF-36	65 (45/85) [0 → 100] [*n* = 824]	65 (45/85) [0 → 100] [*n* = 652]	55 (40/80) [0 → 100] [*n* = 172]	0.015
PDI score	2.1 (0.7/3.7) [0 → 10] [*n* = 825]	2.1 (0.7/3.7) [0 → 10] [*n* = 652]	2.0 (0.8/4.0) [0 → 9] [*n* = 173]	0.575

Results for ordinal data with median (25th/75th percentile) [min → max] or data following Gaussian distribution were presented as mean± SD.

### Lp(a) level and ASCVD

Among the 850 patients who participated in this WalkByLab study, 533 fulfilled the high-risk ASCVD criteria, whereas 317 were healthy controls. Significantly more ASCVD patients than healthy participants presented a high-Lp(a) value (24.2% vs. 14.8%, see Table 2). Both CAD and PAD, as well as CeVD, occurred significantly more often in the high-Lp(a) group: 36.8% of low-Lp(a) level patients were diagnosed with CAD compared to 50% with high Lp(a). A diagnosis of PAD was observed in 45.8% vs. 57.7% of patients with the low-Lp(a) group vs. the high-Lp(a) group, respectively. CeVD was present in 9.5% vs. 17.6% of patients with the low-Lp(a) group vs. the high-Lp(a) group, respectively. Due to the relatively high number of cases of systemic atherosclerosis, including both CAD and concomitant PAD, these were assessed separately. A total of 22.9% of patients with the low-Lp(a) group were diagnosed with combined CAD and PAD compared to 35.4% in the high-Lp(a) group.

**Table 2:** Patient risk factors, disease status, and medication in the low-Lp(a) and high-Lp(a) groups.

Risk factors/ vascular diseases	Overall	Low-Lp(a) group	High-Lp(a) group	*p*
Participants	850			0.001
ASCVD patients	533	75.8% (*n* = 404)	24.2% (*n* = 129)	
Healthy controls	317	85.2% (*n* = 270)	14.8% (*n* = 47)	
CAD	332	36.8% (*n* = 246)	50% (*n* = 86)	0.004
PAD	409	45.8% (*n* = 308)	57.7% (*n* = 101)	0.004
CAD and PAD				0.004
CAD and PAD yes	215	22.9% (*n* = 153)	35.4% (*n* = 62)	
CAD only	118	13.9% (*n* = 93)	14.3% (*n* = 25)	
PAD only	194	23.2% (*n* = 155)	22.3% (*n* = 39)	
CAD and PAD no	316	40% (*n* = 267)	28% (*n* = 49)	
CeVD	91	9.2% (*n* = 60)	17.6% (*n* = 31)	0.007
Diabetes mellitus	198	23.5% (*n* = 158)	22.7% (*n* = 40)	0.920
Hypertension	600	70.1% (*n* = 472)	72.7% (*n* = 128)	0.250
Heart failure	460	53.2% (*n* = 352)	72.8% (*n* = 108)	0.014
Myocardial infarction	136	13.9% (*n* = 92)	24.7% (*n* = 43)	0.001
Smoking				0.728
Current smoker	164	20.7% (*n* = 132)	18.7% (*n* = 32)	
Ex-smoker	210	25.4% (*n* = 162)	28.1% (*n* = 48)	
Never	436	54% (*n* = 345)	53.2% (*n* = 91)	
Medication	Overall	Low-Lp(a) group	High-Lp(a) group	*p*
ACEis	263	29.8% (*n* = 201)	35.2% (*n* = 62)	0.171
Anticoagulation	165	19% (*n* = 128)	21% (*n* = 37)	0.593
ARBs	245	28.3% (*n* = 191)	30.7% (*n* = 54)	0.575
Beta-blockers	372	40.7% (*n* = 274)	55.7% (*n* = 98)	0.001
Clopidogrel	100	10.1% (*n* = 68)	18.2% (*n* = 32)	0.005
Diuretics (alodosterone)	31	3.6% (*n* = 24)	4% (*n* = 7)	0.821
Ezetimibe	63	5.5% (*n* = 37)	14.8% (*n* = 26)	0.001
PCSK9i	4	0.3% (*n* = 2)	1.1% (*n* = 2)	0.191
P2Y12 inhibitors	122	12.6% (*n* = 85)	21% (*n* = 37)	0.008
Rivaroxaban	96	10.5% (*n* = 68)	18.4% (*n* = 28)	0.039
Statins	451	50.4% (*n* = 340)	63.1% (*n* = 111)	0.003

In a logistic regression model with sex, age, hypertension, diabetes, smoking, BMI, and Lp(a) ≥50 mg/dl as independent variables, Lp(a) was significantly associated with ASCVD (OR 2.26, 95% CI 1.32–3.95, *p* = 0.003). LDL-C was excluded from the model due to the high number of missing values. As Lp(a) and LDL-C are uncorrelated (*r* = −0.03, n.s.), this will have little or no impact on the results. In a further logistic regression model, CAD alone was analyzed with statin use, sex, age, hypertension, diabetes, smoking, BMI, and Lp(a) ≥50 mg/dl as independent variables, and Lp(a) was significantly associated with CAD (OR 1.55, 95% CI 1.01–2.37, *p* = 0.043). Furthermore, PAD was analyzed in a logistic regression model, with statin use, sex, age, hypertension, diabetes, smoking, BMI, and Lp(a) ≥50 mg/dl as independent variables, and Lp(a) was significantly associated with CAD (OR 1.61, 95% CI 1.04–2.50, *p* = 0.033).

In a subsequent logistic regression model, statin use, age, hypertension, diabetes, smoking, BMI, and Lp(a) ≥ 50 mg/dl were included as independent variables in the analysis of sex differences. In both sexes, Lp(a) was no longer significantly associated with ASCVD, with OR of 2.55 (95% CI 0.92–7.66, *p* = 0.072) for men and 1.93 (95% CI 0.90–4.17, *p* = 0.090) for women. This lack of significance may be due to lower power with smaller sample sizes.

### Lp(a) and risk factors and mortality

Significantly more patients in the high-Lp(a) group exhibited a higher cardiovascular morbidity status, reflected by the presence of heart failure (72.8% vs. 53.2%, Table 2) and myocardial infarction (24.7% vs. 13.9% *n* = 43). The prevalence of diabetes and hypertension, however, did not seem to differ between the low-Lp(a) and high-Lp(a) groups. As modifiable risk factors, smoking and physical activity showed no relation to Lp(a) levels.

### Lp(a) level and medication

The most common medications were statins (*n* = 451) and antihypertensives such as beta-blockers (*n* = 372), angiotensin-converting enzyme inhibitors (ACEis) (*n* = 262), and angiotensin II receptor blockers (ARBs) (*n* = 245) (Table 2). Significantly more patients with high Lp(a) levels than those with low-Lp(a) levels received statins, ezetimibe, beta-blockers, and P2Y12 inhibitors; see Table 2 for further details. No correlation was found between LDL-C levels, cholesterol, and Lp(a) levels. Clopidogrel and rivaroxaban were significantly more often prescribed in the high-Lp(a) group (18.2%/18.4%) compared to the low-Lp(a) group (10.1%/10.5%).

### Lp(a) and angiological status

A significant increase in atherosclerotic risk status was observed with increasing Lp(a) (Table 3). The percentage of patients with more severe PAD was increased in the high-Lp(a) group. In the high-Lp(a) group, 34% of patients suffered from moderate intermittent claudication compared to only 19% in the low-Lp(a) group. In the high-Lp(a) group, the percentage of patients with severe intermittent claudication was 29.9% (*n* = 29), again exceeding the percentage of patients in the low-Lp(a) group with 21.4% (*n* = 63).

**Table 3:** Detailed angiological status, vascular interventions, and activities.

Angiological status	Low-Lp(a) group	High-Lp(a) group	*P*
PAD Rutherford Stadium classification			0.004
No symptoms or limitations in physical activity	43.9% (*n* = 129)	26.8% (*n* = 26)	
Mild intermittent claudication, Doppler >50 mmHg	12.9% (*n* = 38)	8.2% (*n* = 8)	
Moderate intermittent claudication	19% (*n* = 56)	34% (*n* = 33)	
Severe intermittent claudication, Doppler <50 mmHg	21.4% (*n* = 63)	29.9% (*n* = 29)	
Pain at rest	2% (*n* = 6)	1% (*n* = 1)	
Distal atrophic lesion with acral tissue loss	0.3% (*n* = 1)	0% (*n* = 0)	
Lesion originating proximally	0.3% (*n* = 1)	0% (*n* = 0)	
ABI			0.002
≤0.7	6.3% (*n* = 42)	14.9% (*n* = 26)	
0.7–<0.9	16.8% (*n* = 113)	17.7% (*n* = 31)	
Normal ≥0.9	76.9% (*n* = 516)	67.4% (*n* = 118)	
Number of peripheral vascular interventions			0.007
0	80.7% (*n* = 486)	70.1% (*n* = 115)	
1	6.8% (*n* = 41)	6.7% (*n* = 11)	
2	4.3% (*n* = 26)	6.1% (*n* = 10)	
>2	49 (8.1%)	17.1% (*n* = 28)	
Number of previous coronary interventions			0.015
0	63.2% (*n* = 259)	50.8% (*n* = 63)	
1	20.7% (*n* = 85)	23.4% (*n* = 29)	
2	11.2% (*n* = 46)	13.7% (*n* = 17)	
>2	4.9% (*n* = 20)	12.1% (*n* = 15)	
Family history with CHD			0.014
Yes	33.1% (*n* = 210)	43.5% (*n* = 73)	
No	66.9% (*n* = 425)	56.5% (*n* = 95)	
Sports			0.853
Yes	31.5% (*n* = 193)	32.6% (*n* = 56)	
No	68.5% (*n* = 419)	67.4% (*n* = 116)	

Participants without previous coronary or peripheral vascular intervention were significantly more likely to be in the low-Lp(a) group than in the high-Lp(a) group. In contrast, patients with one, two, or more interventions were significantly more likely to be in the high-Lp(a) group. A positive CVD family history was significantly more often reported in the high-Lp(a) group compared to the low-Lp(a) group (43.5% vs. 33.1%). A lower ABI of ≤0.7 correlated with high Lp(a), whereas a normal ABI was less often recorded in the low-Lp(a) group.

### Examination of Lp(a) levels over the period of 1 year

**Figure 1:**
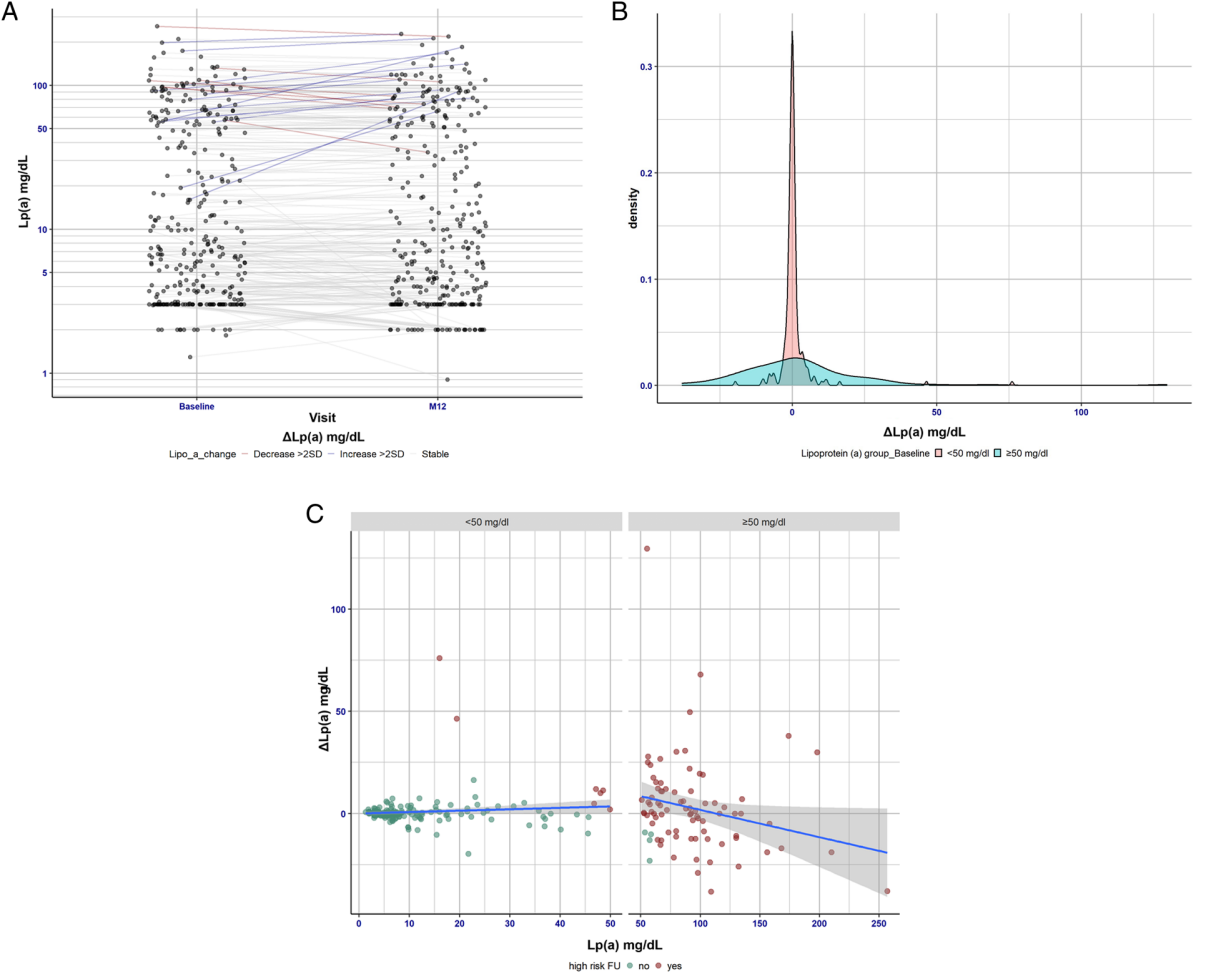
(**A**) Lp(a) changes over the period of 1 year (baseline vs. 12-month follow-up visit). This panel shows the differences between Lp(a) levels at baseline vs. 12-month follow-up. It shows that Lp(a) is very stable over the 1 year period—the median change was 0 mg/dl and the percentile range (25/75) was (−1.0/1.3). Hence, change scores show no clinically relevant variation. Patients showing substantial changes in Lp(a) levels were considered separately. Here, patients with Lp(a) increase above 22 mg/dl [>2 standard deviations (SDs)] were considered to have significant changes. Among the patients examined this way, 7 (4 men, 3 women) showed a reduced Lp(a) value and 13 (8 men, 5 women) showed an increased Lp(a) value over a period of 1 year. (**B**) Visualization of the distribution of the Lp(a) concentration in the high-Lp(a) and low-Lp(a) groups. The delta Lp(a) observation distinguishes between the low-Lp(a) group (<50 mg/dl) and the high-Lp(a) group (≥50 mg/dl). (**C**) Delta difference and concordance/agreement level of Lp(a) in the low- and high-Lp(a) groups using a standard Bland–Altman plot. Patients with Lp(a) levels below 50 mg/dl at baseline have been highlighted in green, and patients with Lp(a) levels exceeding 50 mg/dl at follow-up (12 months) have been highlighted in red. It is evident from the visual representation that only a small number of cases showed Lp(a) changes resulting in a transition from low to high Lp(a) levels and vice versa. Visual representation demonstrated that only a small number of cases showed Lp(a) changes that resulted in transition from low-Lp(a) to high-Lp(a) levels and vice versa (Figure 1C).

In 271 patients, Lp(a) measurements were repeated after 1 year (Figure 1A) and confirmed the stable value despite CVD treatment intensifications. The mean change in the low- and high-Lp(a) patients was only 1 mg/dl, and the mean SD was ±11 mg/dl. Analysis of ΔLp(a) in more detail (Figure 1B) revealed that there are bigger deviations in Lp(a) levels (±39 SD) in the high-Lp(a) group over the period of 1 year. In contrast, in the low-Lp(a) group, Lp(a) hardly changes over a period of 1 year. Visual representation demonstrated that only a small number of cases showed Lp(a) changes that resulted in transition from low-Lp(a) to high-Lp(a) levels and vice versa (Figure 1C).

## Discussion

This study investigated the Lp(a) characteristics of patients at high and low cardiovascular risk within an understudied non-metropolitan patient cohort in Brandenburg, Germany. Cardiovascular patients and healthy controls presenting to the WalkByLab Brandenburg underwent a comprehensive cardiovascular and angiological screening, blood serum analysis, and medical history documentation. This study confirms that Lp(a) levels >50 mg/dl in a non-metropolitan population in Brandenburg are significantly associated with higher rates of ASCVD (CAD, PAD, CeVD) as well as heart failure, myocardial infarction, and a positive family history of CAD. Approximately 20%–25% of the world's population has an Lp(a) level of 50 mg/dl (3); therefore, we chose the threshold value advised by the EAS for our analyses. Also, the BiomCaRE (Biomarkers for Cardiovascular Risk Assessment in Europe) consortium recommended not only focusing on percentile-based analyses, as commonly done in Lp(a) studies, but also emphasizing the clinically significant threshold of 50 mg/dl. They identified a robust association between major cardiovascular events (MCE) and CVD events in seven prospective population-based cohorts across Europe when Lp(a) levels were >50 mg/dl compared to levels <50 mg/dl (15). The cardiovascular risk observed in our Brandenburg cohort confirms these data and aligns with findings from another trial involving patients from Saxony-Anhalt, Germany (15, 16). Elevated Lp(a) levels were exhibited in an extraordinarily high cardiovascular risk population. The high rate of statin treatment in our high-Lp(a) group seems to reflect the focus of physicians on optimizing modifiable risk factors, such as high LDL cholesterol levels, due to the absence of specific anti-Lp(a) treatments (17). However, as we know from the JUPITER trial and others, elevated Lp(a) levels significantly contribute to the residual risk of cardiovascular disease, even in participants with very low LDL-C levels resulting from high-dose aggressive statin treatment (8, 18). Although current therapies are limited in their ability to effectively lower Lp(a) levels, new nucleic acid-based treatments (small interfering RNAs), such as pelacarsen (19) and olpasiran (20), show that a significant reduction in Lp(a) levels is possible. In a study of pelacarsen, baseline Lp(a) levels varied between 204.5 and 246.6 nmol/L across six groups, and the application of APO(a)-LRx resulted in dose-dependent reductions, exhibiting mean percent decreases ranging from 35% to 80%, in contrast to a 6% reduction observed with the placebo (19). In a Phase 1 trial involving healthy participants, muvalapline, an orally administered small-molecule inhibitor of Lp(a) formation, exhibited safety, tolerability, and a dose-dependent reduction in Lp(a) levels of up to 65%, without affecting plasminogen activity (21). It was recently reported by Paige et al. and others that diabetes risk is inversely associated with Lp(a) concentration, with a higher risk of type-2 diabetes at low Lp(a) concentrations (22, 23). Our result in regard to diabetes mellitus and Lp(a) levels did not show any association. Future studies will evaluate the cardiovascular outcomes and safety of these Lp(a)-lowering therapies, with a focus on the possible recurrence of diabetes in patients with very low Lp(a) levels (3). However, while significant reductions in Lp(a) levels may lead to improvements, it is important to note that not all cardiovascular risks may be completely eliminated. Other risk factors, lifestyle choices, and genetic predispositions may still contribute to overall cardiovascular health. Therefore, while reducing elevated Lp(a) is a positive step, comprehensive cardiovascular risk management may involve addressing multiple factors for optimal prevention and treatment.

Our data also support previous scientific evidence of a causal role of Lp(a) in the development of heart failure (24, 25). In this study, we also showed higher numbers for hypertension in the high-Lp(a) group. In this context, the MESA study by Rikhi et al., with multiethnic cohorts published recently, investigated the threshold of ≥50 mg/dl Lp(a) in hypertension and demonstrated that hypertension occurs more often in individuals with high Lp(a) (26).

Within the non-metropolitan patient cohort in Brandenburg, our data reveal a significant trend, with individuals having a family history of chronic heart disease (CHD) being more prevalent in the high-Lp(a) group, suggesting a potential link between familial CHD predisposition and elevated Lp(a) levels. Our data suggest that at least assessing the family medical history of CHD can serve as an easy prescreening tool for selecting suitable patients for a once-in-life Lp(a) measurement, particularly in the context of non-standardized Lp(a) assessments. This may aid in identifying individuals who might benefit from a focused assessment of Lp(a) levels, potentially enhancing the implementation of targeted preventive measures for those at elevated risk. This prescreening tool is not intended as a standalone diagnostic method but rather as a preliminary step in selecting suitable patients for a focused Lp(a) assessment. Such an approach may contribute to more efficient resource allocation, especially in regions with limited healthcare resources.

Our 1-year follow-up cohort confirmed the known fact that Lp(a) levels do not change over time with available treatment options (27). The fact that Lp(a) levels remain relatively unchanged over the lifetime of a person is an influential reason why, to date, Lp(a) has received little attention in clinical practice (27).

In this study, the higher numbers of cardiovascular medications such as antihypertensives or clopidogrel can be attributed to the increased general cardiovascular risk of the high-Lp(a) group.

## Conclusion

This study demonstrates that individuals within a Brandenburg non-metropolitan cohort with high Lp(a) levels express a significantly higher cardiovascular risk and comorbidity profile than similar individuals with low Lp(a) levels. Results indicate that high Lp(a) levels serve as an independent predictor of cardiovascular disease in participants from Brandenburg, Germany. Our results on low and high Lp(a) levels among patients with and without cardiovascular disease provide awareness and inform strategies aimed at improving diagnostic and therapeutic options for patients at risk of ASCVD.

## Data Availability

The original contributions presented in the study are included in the article/supplementary material, further inquiries can be directed to the corresponding author.
